# Great expectations

**DOI:** 10.1590/S1516-31802012000100001

**Published:** 2012-02-13

**Authors:** Paulo Manuel Pêgo-Fernandes, Benoit Jacques Bibas

**Affiliations:** I MD, PhD. Associate Professor, Discipline of Thoracic Surgery, Instituto do Coração (InCOR), Hospital das Clínicas (HC), Faculdade de Medicina da Universidade de São Paulo (FMUSP), São Paulo, Brazil.; II MD. Trainee Physician in Tracheal Surgery and Therapeutic Respiratory Endoscopy, Discipline of Thoracic Surgery, Hospital das Clínicas, Faculdade de Medicina da Universidade de São Paulo.

In November 2011, the new board of the Associação Paulista de Medicina (APM) took office for the three-year period 2011-2014, under the presidency of Dr. Florisval Meinão. Among the targets of the new board, strengthening and expansion of the São Paulo Medical Journal (SPMJ) are priorities. It is our intention, and duty, to establish closer contact with specialist societies and offer the SPMJ as a vehicle for scientific publication at international level. A large proportion of the specialist societies do not have scientific journals indexed in the Medline database, and the SPMJ could be the ideal means for publishing Brazilian scientific studies that are produced. It is known that a sizable portion of the scientific pro duction of specialist Brazilian congresses does not reach publication in scientific journals.^1^^,^^2^ Therefore, it is up to us, i.e. authors, editors and editorial board members, to push forward publication of our studies. Only through hard work together will we be able to achieve the desired goal of strong and effective national science.

Evidence-based medicine is increasingly important, both within Brazilian medicine and internationally. The SPMJ has the objective of publishing papers from all areas of medicine, with a focus on evidence-based medicine. Original articles, systematic reviews, cases reports and Cochrane highlights are published. The SPMJ has now been indexed in the ISI Journal Citation Reports database for two years.^1^^,^^2^ This has consolidated our journal among the highest-regarded in the country, and has given it visibility worldwide. In fact, we are now receiving a variety of studies from researchers outside of Brazil, and this number is on the rise.^2^ This is the context within which we intend to expand our actions further.

Where Brazilian periodicals are heading, and their future, was a topic greatly discussed by the outgoing board over the last three years, and it continues to be a matter of con cern for the Brazilian scientific community.^3^^,^^4^^,^^5^^,^^6^ It is known that Brazilian researchers prefer to publish their material in foreign journals, rather than in Brazilian journals.^7^ Through making this choice, the standing of the postgraduate program to which they are linked is improved, their articles achieve higher impact factors and their H index is increased. How ever, the search for greater visibility and quality of national production can be assessed not only through the articles, but also through greater qualification of our periodicals, so that they are recognized internationally.^7^ The impact factor published by the Journal Citation Reports (Thomson Reuters) pro vides an (imperfect) measurement of the quality of periodicals, but does not in any way measure the quality of each article published in a given journal.^1^^,^^5^ Theoretically, it would be much more appropriate to count the number of citations received for each article, as a measurement of its quality. However, such a procedure would have limited applicability for evaluating the scientific production of postgraduate students and their supervisors.^5^ The particular features of each area of interest or each specialty need to be taken into con sideration and respected. Support and incentives need to be expanded: these could come in the form of bursaries for editors, financial support for publication, greater visibility for Brazilian periodicals abroad and criteria for qualitative classification that are more objec tive and comprehensive.^8^

We take the opportunity of the start of the new administration to give thanks to everyone who in some way has contributed towards advancing the SPMJ. We also express our gratitude to editorial board members, reviewers and authors, who have been mov ing forward with us towards strengthening Brazilian science. The São Paulo Medical Journal has, through its editors, editorial board members and reviewers, been working continually to develop and improve the journal. We begin the year 2012 with great expecta tions, with prospects of growth, expansion, science and a lot of work. We are sure that the three-year period 2011-2014 will include many achievements and much delight.

A happy 2012 to everyone!
